# The inclusion membrane protein IncS is critical for initiation of the *Chlamydia* intracellular developmental cycle

**DOI:** 10.1371/journal.ppat.1010818

**Published:** 2022-09-09

**Authors:** María Eugenia Cortina, R. Clayton Bishop, Brittany A. DeVasure, Isabelle Coppens, Isabelle Derré

**Affiliations:** 1 Department of Microbiology, Immunology, and Cancer Biology, University of Virginia School of Medicine, Charlottesville, Virginia, United States of America; 2 Department of Molecular Microbiology and Immunology, Johns Hopkins School of Public Health, Baltimore, Maryland, United States of America; University of Cambridge, UNITED KINGDOM

## Abstract

All *Chlamydia* species are obligate intracellular bacteria that undergo a unique biphasic developmental cycle strictly in the lumen of a membrane bound compartment, the inclusion. *Chlamydia* specific Type III secreted effectors, known as inclusion membrane proteins (Inc), are embedded into the inclusion membrane. Progression through the developmental cycle, in particular early events of conversion from infectious (EB) to replicative (RB) bacteria, is important for intracellular replication, but poorly understood. Here, we identified the inclusion membrane protein IncS as a critical factor for *Chlamydia* development. We show that a *C*. *trachomatis* conditional mutant is impaired in transition from EB to RB in human cells, and *C*. *muridarum* mutant bacteria fail to develop in a mouse model of *Chlamydia* infection. Thus, IncS represents a promising target for therapeutic intervention of the leading cause of sexually transmitted infections of bacterial origin.

## Author summary

The identification and characterization of bacterial factors that are essential, or highly important, to the intracellular life style of obligate intracellular bacterial pathogens, for which limited genetic tools exist, present unique challenges. Here, we offer a blueprint to generate conditional mutants in *Chlamydia trachomatis* and *Chlamydia muridarum*, by combining existing genetic tools for *Chlamydia*, including an inducible promoter that is responsive in a murine model of *Chlamydia* infection. As a proof of concept, we present a conditional mutant in a *Chlamydia* inclusion membrane protein and show that it is impaired in the early stages of the developmental cycle. Our study constitutes a major methodological advance to the study of *Chlamydia* and furthers our understanding of the bacterial factors contributing to progression through the developmental cycle.

## Introduction

*C*. *trachomatis* is the leading cause of bacterial sexually transmitted diseases. 1.7 million cases are reported in the United States annually, and there are an estimated 131 million new cases each year worldwide [1, 2]. Infections are often asymptomatic, especially in women, which increases the risks of transmission to sex partners. In addition, if left untreated, cervical infections ascend to the upper genital tract resulting in tissue inflammation leading to pelvic inflammatory disease, ectopic pregnancy and infertility [3]. Vaccines are not available [4, 5], and because of short-lived protective immunity from infection, reinfection rates are high and contribute to the aggravation of the long-term sequelae associated with infection.

All *Chlamydia* species are obligate intracellular bacteria that replicate exclusively in epithelial cells within a membrane-bound compartment, termed the inclusion, and via a unique biphasic developmental cycle [6]. The first 8 hours are especially critical in establishing a replicative niche because invaded, infectious, morphologically small, but non-replicative elementary body (EB) must transition to replicative, morphologically larger, albeit non-infectious, reticulate body (RB). Concomitantly, the nascent inclusions traffic along microtubules to the microtubule organizing center (MTOC) [7]. Bacterial transcription and translation are both required during the early events of the developmental cycle [7]; however, the specific mechanism(s) by which EB transition to RB is unclear.

Inclusion membrane proteins (Inc) are *Chlamydia*-specific Type III secretion effector proteins that are embedded into the inclusion membrane and proposed to facilitate inclusion maturation and inclusion membrane stability [8, 9]. *Chlamydia* encodes 50–100 putative Inc proteins, some, but not all, are conserved among species [10–13]. A comprehensive list of Inc-host protein interactions was identified, and in part validated [9, 14]. Nevertheless, the specific functions of many Inc proteins remain elusive, due much to a lag in *Chlamydia* genetics [15]. Recent progress led to the identification of a few *C*. *trachomatis inc* mutants with *in vitro* and/or *in vivo* defects [12, 16–18]. However, none of them have been implicated in the early stages of the developmental cycle. Additionally, in light of the obligate intracellular lifestyle of the pathogen, the overall viability of the above-mentioned *C*. *trachomatis* mutants and the fact that they were identified in the first place, indicate that the corresponding Inc proteins are not essential.

Essential virulence factors, preferably common to all *Chlamydia* species, would be ideal drug targets to limit the spread of the bacteria. They remain unknown, and their identification is challenging. Without the option of cultivating the bacteria in axenic medium, *Chlamydia* mutants with severe intracellular developmental defects cannot be propagated and therefore would be missed using current targeted or unbiased genetic approaches. Here, through the use of *C*. *trachomatis* and *C*. *muridarum* conditional mutants, we report a conserved Inc protein that plays a critical role in initiation the *Chlamydia* developmental cycle *in vitro* and *in vivo*.

## Results

### Generation of a *C*. *trachomatis incS* conditional mutant overcomes IncS requirement in cell culture

**Fig 1:**
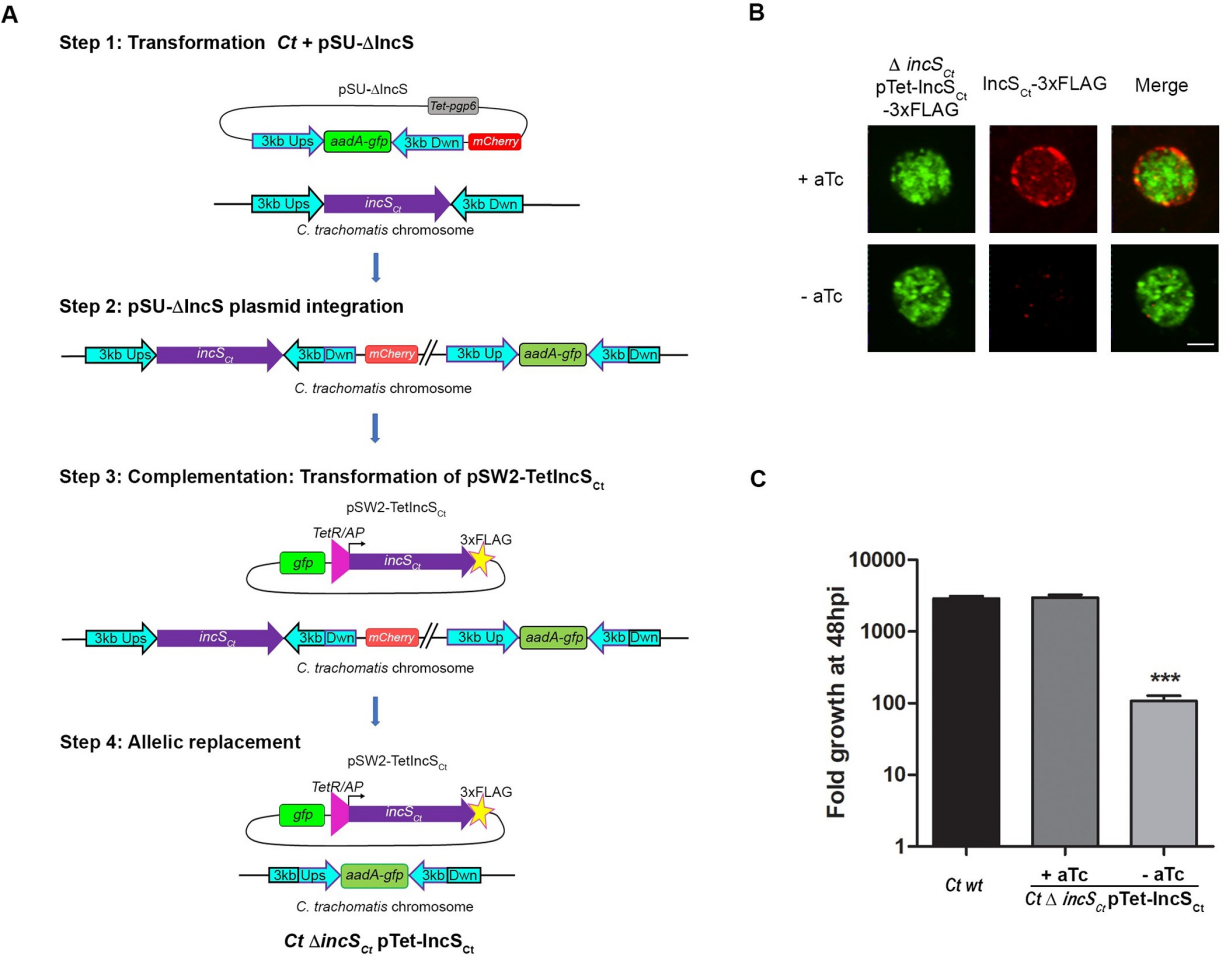
A *Chlamydia trachomatis incS* conditional mutant displays a severe growth defect in cell culture. (A) Schematic representation of the modified 4-step FRAEM strategy used to generate a *C*. *trachomatis incS* conditional mutant (*Ct ΔincS*_*Ct*_ pTet-IncS_Ct_). Step1: Transformation of wild-type *C*. *trachomatis*, containing an intact chromosomal *incS*_*Ct*_ ORF (purple), with pSU-Δ*incS* harboring ~3kb homology sequence upstream and downstream of the *incS* ORF (3kb Ups and 3kb Dwn, respectively, cyan) flanking the *aadA-gfp* selection cassette (green), mCherry (red), and the plasmid maintenance ORF *pgp6* under the control of the aTc inducible promoter (gray). Step 2: Integration of pSU-Δ*incS* in the *C*. *trachomatis* chromosome upstream or downstream (shown here) of the *incS* ORF, via a single recombination event, resulting in a strain displaying dim green and red fluorescence. Step 3: Transformation of the strain resulting from step 2 with pSW2-Tet-IncS_Ct_, a complementation plasmid encoding GFP (green) and a 3x-FLAG tagged (yellow star) allele of IncS_Ct_ (purple) under the control of the aTc inducible promoter (TetR/AP, pink triangle). Step 4: Expression of IncS from the complementation plasmid, allows for allelic replacement of the chromosomal *incS* ORF with the *aadA-gfp* cassette and loss of mCherry, via a second event of recombination within the 3 kb upstream sequence. (B) Three-dimensional confocal micrographs of HeLa cells infected for 24h with the *C*. *trachomatis ΔincS*_*Ct*_ conditional mutant expressing GFP (*ΔincS*_*Ct*_ pTet-IncS_Ct_-3xFLAG, left panels, green) in the presence (+aTc, top panels) or the absence (-aTc, bottom panels) of aTc and stained with anti-FLAG antibody (IncS_Ct_-3xFLAG, middle panels, red). The merge is shown on the right. Scale bar: 5μm. (C) Fold growth of wild-type *C*. *trachomatis* (*Ct wt*) and the *C*. *trachomatis ΔincS*_*Ct*_ conditional mutant (Δ*incS*_*Ct*_ pTet-IncS_Ct_) in HeLa cells at 48h pi compared to 0h pi in the presence (+aTc) or absence (-aTc) of aTc. Data are mean ± SEM, three combined experiments, One-way ANOVA, Tukey’s Multiple Comparison Test, *** P<0.001 (-aTc vs + aTc or wt).

To investigate the role of the *C*. *trachomatis* Inc protein CTL0402, we sought to generate a *ctl0402* mutant using TargeTron or FRAEM (fluorescence reported allelic exchange mutagenesis) [19, 20]. After several unsuccessful attempts, we hypothesized that *ctl0402* may be required for proper bacterial development in cell culture. CTL0402 was renamed IncS_Ct_, and we developed a 4-step strategy to generate a *ΔincS*_*Ct*_ conditional FRAEM mutant (Fig 1A). *C*. *trachomatis* wild-type was first transformed with the pSU-ΔincS plasmid which encodes the *aadA*-*gfp* selection cassette surrounded by chlamydial DNA corresponding to ~3 kb of genomic sequence flanking the *incS*_*Ct*_ ORF, mCherry under the control of a constitutive promoter, and the *Chlamydia* plasmid maintenance ORF *pgp6* under the control of the anhydrotetracycline (aTc) inducible promoter (Fig 1A Step 1). GFP- and mCherry-positive transformants were selected in the presence of 500 μg/ml of Spectinomycin (Spec) and 50 ng/ml of aTc. Chromosomal integration of pSUΔincS via a single event of recombination within a 3kb region upstream, or downstream, of the *incS*_*Ct*_ ORF, indicated by dim green and red fluorescence, was accomplished by cultivating the transformants in the absence of aTc for multiple passages at low MOI (~0.2) (Fig 1A Step 2). The resulting strain was transformed with pSW2-TetIncS_Ct_, a complementation plasmid carrying a 3xFLAG tagged allele of IncS_Ct_ under the control of the aTc inducible promoter, in the presence of 0.5 ng/ml aTc, 500 μg/ml Spec and 1U Penicillin G (Fig 1A Step 3). The aTc concentration was empirically determined based on the detection of low levels of IncS_Ct_-3xFLAG at the inclusion membrane, when expressed in wild-type *C*. *trachomatis* (not shown). After several passages at a low MOI in the presence of aTc, Spec and Penicillin G, a second event of recombination occurred in some bacteria, leading to the allelic replacement of the *incS*_*Ct*_ ORF with an *aadA-gfp* cassette and the loss of mCherry expression (Fig 1A Step 4). Once the population reached 50% of GFP-positive and mCherry-negative inclusions, the bacteria were plaque purified. The resulting *C*. *trachomatis* strain (*Ct ΔincS*_*Ct*_ pTet-IncS_Ct_) is referred to as the *ΔincS*_*Ct*_ conditional mutant. Three independent clones were amplified, validated as follow, and shown to display the same phenotype. Allelic exchange was confirmed by PCR (S1 Fig). The conditional expression and inclusion localization of IncS_Ct_ was validated by immunofluorescence (Fig 1B). To determine if the lack of IncS affected the production of infectious progeny, cells were infected with the *ΔincS*_*Ct*_ conditional mutant in the presence or absence of aTc for 48h pi, and recovered infectious bacteria were enumerated on a fresh monolayer of cells (Fig 1C). Wild-type *C*. *trachomatis* expressing mCherry constitutively was used as a positive control. In the absence of aTc, the *ΔincS*_*Ct*_ conditional mutant presented a 1.5-log reduction in the number of infectious progenies, compared to the complemented strain, which yielded similar number of infectious progenies as the wild-type strain. Thus, as predicted by our failed attempts to generate a *C*. *trachomatis incS* mutant, the lack of IncS leads to a severe growth defect, and IncS requirement in cell culture can be bypassed by generating a conditional mutant.

### A *C*. *trachomatis incS* mutant fails to initiate the developmental cycle

**Fig 2:**
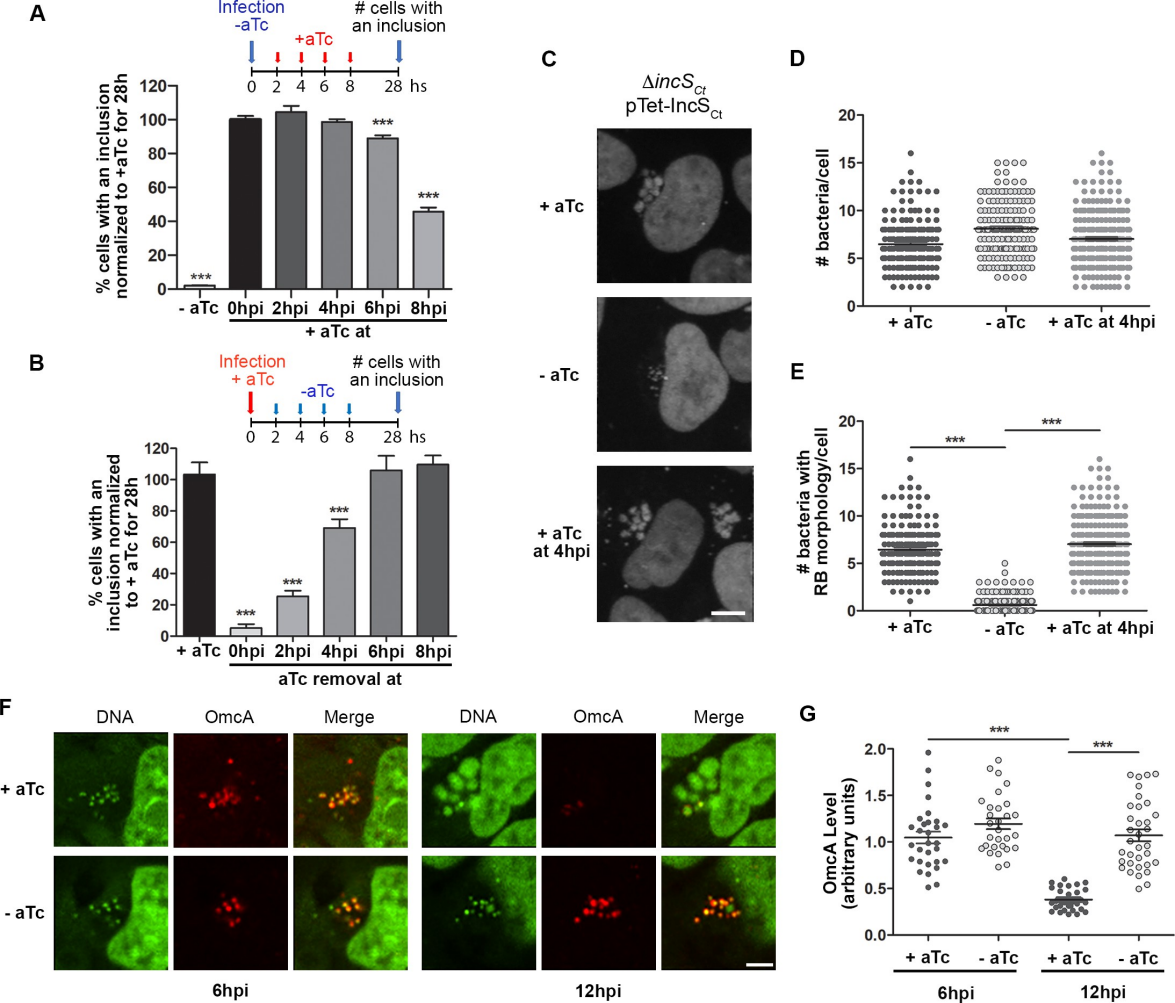
Early developmental defect of a *Chlamydia trachomatis incS* conditional mutant. (A-B) Percentage of cells displaying an inclusion at 28h pi after infection with *C*. *trachomatis ΔincS*_*Ct*_ conditional mutant. Infections were initiated in the absence (A) or presence (B) of aTc, and aTc was added (A) or removed (B) at the indicated time. Infections carried out in the absence (A, -aTc) or presence (B, +aTc) served as controls. Data are mean ± SEM normalized to 0h (A) or +aTc (B), one representative experiment of n = 3, One-Way ANOVA, Dunnett’s Multiple Comparison, *** P<0.001. (C) Three-dimensional confocal micrographs of HeLa cells infected with *C*. *trachomatis ΔincS*_*Ct*_ conditional mutant at an MOI of 10 for 12h in the presence (+aTc) or absence (-aTc) of aTc, or in absence of aTc for the first 4h only (+aTc 4h pi) and stained the DNA dye 7AAD-red. Scale bar: 5μm. (D-E) Quantification of number of bacteria per cell (D) and the number of bacteria with an RB morphology (E), as shown in (C). Each data point represents an infected cell, the mean ± SEM of one representative experiment of n = 3 is shown, One-Way ANOVA, Tukey’s Multiple Comparison, *** P<0.001. (F) Representative single plane confocal micrographs of HeLa cells infected with *C*. *trachomatis ΔincS*_*Ct*_ conditional mutant for 6h (left panels) or 12h (right panels) in the presence (+aTc) or absence (-aTc) of aTc and stained with the EB-specific marker OmcA (red) and the DNA dye SytoxGreen (green). Scale bar: 5μm. (G) Quantification of the levels of OmcA associated with perinuclear bacteria as shown in (F). Each data point represents an infected cell, the mean ± SEM of one representative experiment of n = 3 is shown, One-Way ANOVA, Tukey’s Multiple Comparison, *** P<0.001.

We next sought to investigate the nature of the growth defect of the *ΔincS*_*Ct*_ mutant by inducing or terminating complementation at different times post infection (Fig 2). Cells were infected with the *C*. *trachomatis ΔincS*_*Ct*_ conditional mutant at an MOI of 1 in the absence (Fig 2A) or the presence of aTc (Fig 2B). Infections were synchronized by centrifugation and extensive washes of the monolayers. At 2, 4, 6 or 8h pi, the medium was replaced with 0.5ng/ml aTc (Fig 2A) or medium without aTc (Fig 2B), and the number of infected cells was quantified at 28h pi. The substantial decrease in infectious progeny observed at 48h pi (Fig 1C) correlated with a drastic reduction in the number of infected cells harboring an inclusion at 28h pi (Fig 2A -aTc). Additionally, complementation induction (Fig 2A) or termination (Fig 2B) at different times post infection revealed that *incS* expression was required during the early stages of the developmental cycle, more specifically the first 6-8h, a time at which transition from EB to RB occurs.

To further investigate the intracellular localization and morphology of the *ΔincS*_*Ct*_ mutant bacteria during the early stages of the developmental cycle, cells were infected with the *C*. *trachomatis ΔincS*_*Ct*_ conditional mutant at an MOI of 10 with or without aTc, or with aTc added beginning at 4h pi. Infections were synchronized as described above. Infected cells, stained with a DNA dye, were analyzed by confocal microscopy at 12h pi. EB- and RB-like bacteria were identified based on the size of their respective DNA signal (small and large, respectively) and the number of total and large RB-like intracellular bacteria that clustered at the MTOC were determined (Fig 2C–2E). Regardless of complementation, by 12h pi, a similar number of intracellular bacteria clustered at the MTOC (Fig 2C and 2D). However, the number of bacteria with an RB morphology was significantly reduced in the absence of complementation (Fig 2C and 2E). This phenotype was reverted by complementation at 4h pi. These results suggested that, in cell culture, the *C*. *trachomatis ΔincS*_*Ct*_ mutant may be compromised in transitioning from EB to RB.

To more directly assess if the *C*. *trachomatis ΔincS*_*Ct*_ mutant fails to transition from EB to RB, we performed transmission electron microscopy (TEM) for morphological analysis of the bacteria at 12h pi (S2 Fig). TEM allows to distinguish between EB and RB based on differences in size and electron (e)-density, with EB appearing as small e-dense (black) cocci of ~250 nm in diameter and RB as less e-dense (grey) cocci of ~1 μm in diameter, respectively. Additionally, bacteria transitioning from EB to RB form an Intermediate Body (IB) appearing as small grey cocci of ~500 nm in diameter with a darker center corresponding to condensed chromosomal material. TEM section of inclusions harboring the complemented *ΔincS*_*Ct*_ mutant all contained dividing RBs (S2 Fig, +aTc 12hpi), while EB- or IB-containing inclusions were not observed under this condition. In comparison, while some RB-containing inclusions were observed in cells infected with the *ΔincS*_*Ct*_ mutant, a notable number of inclusions contained bacteria with a size and morphology reminiscent of IBs (S2 Fig, -aTc 12hpi). We estimated that approximately one third of the *ΔincS*_*Ct*_ mutant inclusions contained RBs, one third contained EBs, and one third contained IBs (not shown).

As a complementary approach, we used antibodies against the EB-specific OmcA protein [21] to perform quantitative confocal analysis of the presence of EBs in cells infected with the *C*. *trachomatis ΔincS*_*Ct*_ conditional mutant with or without aTc (Fig 2F and 2G). Our quantification method is detailed in S3 Fig. At 6h pi, complemented and *ΔincS*_*Ct*_ mutant bacteria clustered at the MTOC displayed equivalent levels of OmcA. However, at 12h pi, the complemented *ΔincS*_*Ct*_ mutant bacteria were OmcA-negative, whereas the *ΔincS*_*Ct*_ mutant bacteria remained OmcA-positive, further supporting that by 12h pi *ΔincS*_*Ct*_ mutant bacteria retained EB-like characteristics.

Collectively, these results indicate that, in cell culture, the severe growth defect of the *C*. *trachomatis ΔincS*_*Ct*_ mutant occurs early in the developmental cycle, most likely due to failure of the bacteria to transition from EB to RB.

### IncS requirement to establish a productive infection in cell culture is conserved in *C*. *muridarum*

**Fig 3:**
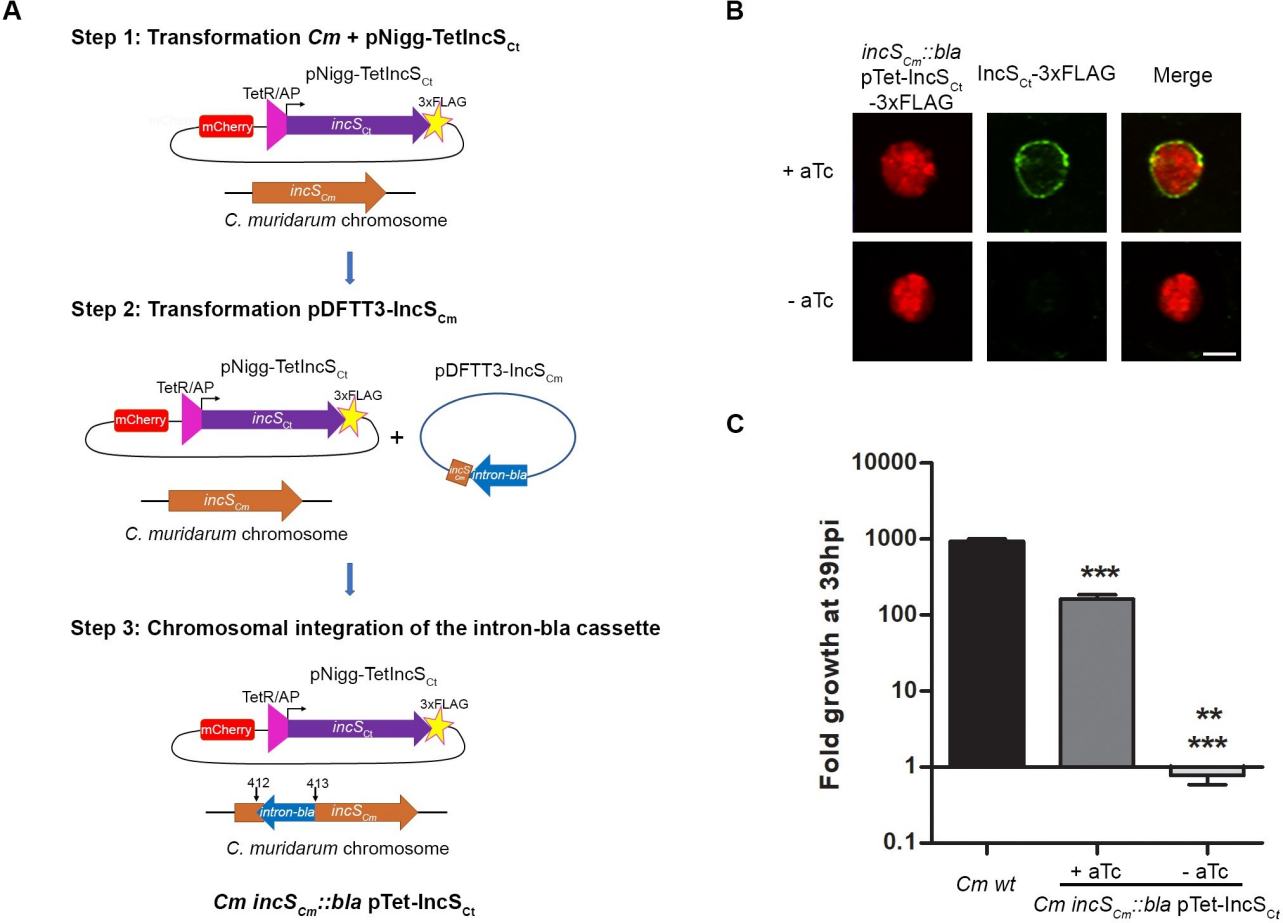
*Chlamydia muridarum incS* conditional mutant displays a severe growth defect in cell culture. (A) Schematic representation of the modified 3-step Targetron strategy used to generate a *C*. *muridarum incS*_*Cm*_ (*tc0424*) conditional mutant (*Cm incS*_*Cm*_::*bla* pTet-IncS_Ct_). Step1: Transformation of wild-type *C*. *muridarum*, containing an intact chromosomal *incS*_*Cm*_ ORF (brown), with pNigg-Tet-IncS_Ct_, a complementation plasmid encoding mCherry (red) and a 3x-FLAG tagged (yellow star) allele of IncS_Ct_ (purple) under the control of aTc inducible promoter (TetR/AP, pink triangle). Step 2: Transformation of the strain resulting from step 1 with pDFTT3-IncS_Cm_ harboring genetic elements to facilitate the insertion of a group II intron and the ß-lactamase gene at nucleotide position 412 of the *incS*_*Cm*_ ORF (*intron-bla*, blue arrow). Step 3: Expression of IncS_Ct_ from the complementation plasmid, allows for integration of the *intron-bla* cassette and interruption of the *incS*_*Cm*_ ORF. (B) Single plane confocal micrographs of HeLa cells infected for 18h with the *Chlamydia muridarum ΔincS*_*Cm*_::*bla* conditional mutant expressing mCherry (*incS*_*Cm*_::*bla* pTet-IncS_Ct_-3xFLAG, left panels, red) in the presence (+aTc, top panels) or the absence (-aTc, bottom panels) of aTc and stained with anti-FLAG antibody (IncS_Ct_-3xFLAG, middle panels, green). The merge is shown on the right. Scale bar: 5μm. (C) Fold growth of wild-type *C*. *muridarum* (*Cm wt*) and the *Chlamydia muridarum ΔincS*_*Cm*_::*bla* conditional mutant (*incS*_*Cm*_::*bla* pTet-IncS_Ct_) in HeLa cells at 39h pi compare to 0h pi in the presence (+aTc) or absence (-aTc) of aTc. Data are mean ± SEM, three combined experiments, One-way ANOVA, Tukey’s Multiple Comparison Test, ** P<0.01 (-aTc vs + aTc); *** P<0.001 (-aTc vs wt).

*C*. *trachomatis* does not establish a productive infection in mice and is rapidly cleared from the murine genital tract [22]; however, upon infection with the murine-adapted species *C*. *muridarum*, mice do recapitulate hallmarks of *Chlamydia* infections observed in women [23]. Therefore, to investigate the role of IncS *in vivo*, we generated a *C*. *muridarum incS*_*Cm*_ (*tc0424*) conditional mutant. FRAEM is not available for *C*. *muridarum*, therefore we developed a 3-step TargeTron-based approach (Fig 3A). *C*. *muridarum* wild-type was first transformed with pNigg-TetIncS_Ct_ (Fig 3A Step 1). Following 3 passages in the presence of 500 μg/ml Spec, transformants were plaque purified and transformed with pDFTT3-IncS_Cm_ (Fig 3A Step 2). The chromosomal integration of the intron/β-lactamase (*bla*) resistance cassette at nucleotide position 412 of the *incS* ORF was selected for in the presence of 1 ng/ml aTc, 500 μg/ml of Spec and 1U PenG. The aTc concentration was empirically determined based on the detection of low levels of IncS_Cm_-3xFLAG at the inclusion membrane, when expressed in wild-type *C*. *muridarum* (not shown). The resulting *C*. *muridarum* strain (*Cm incS*_*Cm*_::*bla* pTet-IncS_Ct_) is referred to as the *incS*_*Cm*_::*bla* conditional mutant. Three independent plaque purified clones were amplified, validated as follow, and shown to display the same phenotype. Intron integration was confirmed by PCR and sequencing (S4 Fig). The conditional expression and inclusion localization of IncS_Ct_ was validated by immunofluorescence (Fig 3B). To determine if inactivation of *incS*_*Cm*_ led to a growth defect in *C*. *muridarum*, cells were infected with the *incS*_*Cm*_::*bla* conditional mutant in the presence or absence of aTc, and the production of infectious progeny was assessed at 39h pi (Fig 3C). Wild-type *C*. *muridarum* expressing mCherry constitutively was used as a positive control. In the absence of complementation, the *incS*_*Cm*_::*bla* conditional mutant barely produced any infectious progeny, and displayed a 2- and 3-log reduction compared to the complemented and wild-type strains, respectively. Addition of aTc resulted in robust, yet partial complementation, with the complemented *incS*_*Cm*_::*bla* mutant displaying a 1-log reduction in infectious progeny production compared to the wild-type strain. Thus, the critical role of IncS in establishing a productive infection in cell culture is conserved between *C*. *trachomatis* and *C*. *muridarum*.

### IncS is required to initiate inclusion development *in vivo*

**Fig 4:**
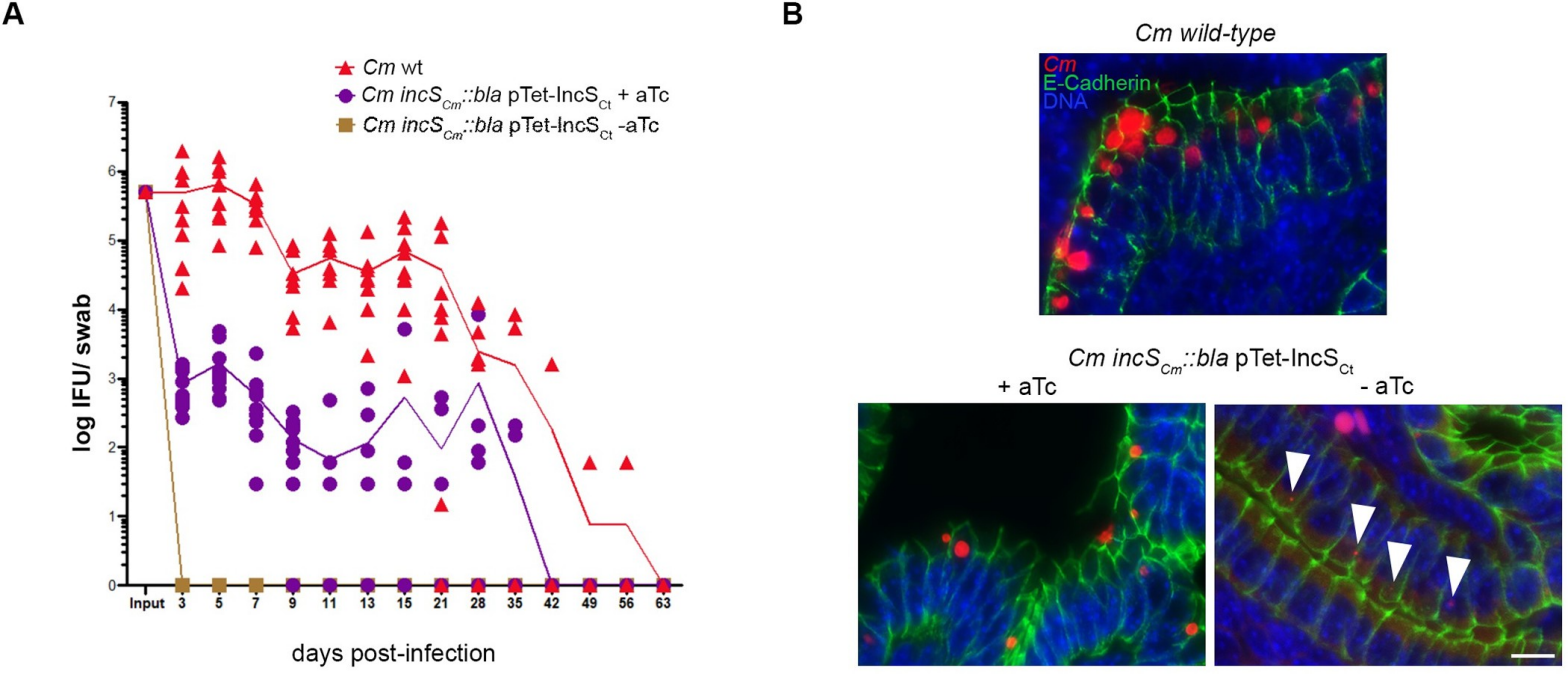
*In vivo* developmental defect of a *Chlamydia muridarum incS* conditional mutant. (A) Infectious progeny recovered at the indicated time from vaginal swabs after intravaginal inoculation of C3H/HeJ mice with wild-type *C*. *muridarum* (red triangles), or the *incS*_*Cm*_::*bla* mutant complemented (purple circles) or not (brown squares) by addition of aTc to the drinking water. Each data point represents a mouse. Combined results from 2 independent experiments with 5 mice per group is shown. Mixed effect analysis with multiple comparisons and Tukey corrections: days 3–9 pi P<0.0001 (all groups), day 11 pi P = 0.0464 (complemented vs mutant), day 13 pi not significant (complemented vs mutant). (B) Fluorescence microscopy images of paraffin sections of endometrial epithelium of C3H/HeJ mice transcervically infected for 8h with wild-type *C*. *muridarum* (top panel), or the *incS*_*Cm*_::*bla* mutant complemented (bottom left panel) or not (bottom right panel, arrowheads indicate intracellular bacteria) by addition of aTc to the drinking water and stained for DNA (blue), E-Cadherin (green) and *C*. *muridarum* (red). One representative mouse shown. Scale bar: 10μm.

To determine if IncS is required for successful infection *in vivo*, C3H/HeJ female mice were intravaginally infected, and vaginal shedding was monitored every other day by swabbing the vaginal vault and enumeration of live infectious bacteria (Fig 4A). In the absence of complementation, mice infected with the *incS*_*Cm*_::*bla* conditional mutant did not shed infectious bacteria, compared to the sustained vaginal shedding of mice infected with wild-type bacteria. Mutant complementation, by addition of aTc in the drinking water of infected mice, led to a significant and sustained bacterial shedding, although shedding was not restored to wild-type levels. To further investigate inclusion development *in vivo*, mice were transcervically infected for 8h with wild-type bacteria, or with the *C*. *muridarum incS*_*Cm*_::*bla* conditional mutant in the presence or absence of aTc in the drinking water. The genital tracts were excised, and infected cells were visualized on paraffin sections by *in situ* labelling of the bacteria and immunostaining of endometrial epithelial cells (Fig 4B). Large inclusions containing multiple bacteria were observed in the endometrial epithelium of mice infected with wild-type bacteria or the complemented mutant (+aTc). In line with the partial complementation of the vaginal shedding phenotype (Fig 4A), inclusions were smaller with the complemented strain than with wild-type bacteria. In contrast, in the absence of complementation (-aTc), infected endometrial cells only displayed single bacterium-containing inclusions (Fig 4B, arrowheads). Thus, the *C*. *muridarum incS*_*Cm*_::*bla* conditional mutant displays a severe, yet complementable, colonization defect *in vivo*, which can be attributed to a defect in inclusion development.

## Discussion

### Identification of essential genes in obligate intracellular pathogens

Inactivation of essential genes is lethal and therefore prevents the amplification and isolation of the corresponding mutant. To overcome this challenge, we combined existing genetics tools for *Chlamydia* to generate targeted *C*. *trachomatis* and *C*. *muridarum* conditional mutants by providing an inducible wild-type allele of the target gene in trans from a complementation plasmid, prior to replacing and interrupting the chromosomal allele with a selection cassette, respectively. Conditional knock-down *C*. *trachomatis* mutants, where expression of the gene of interest is downregulated using the CRISPRi methodology have been described before[24]; however, to our knowledge, this is the first report of a *Chlamydia* conditional knock-out mutant. Another major advance resulting from our study is the conditional expression of the complementation allele *in vivo*. Systemic delivery of inducers, such as doxycycline, is commonly used to generate inducible transgenic mice [25]. We have adapted this approach and shown that administration of aTc in the drinking water of mice was sufficient to yield enough inducer throughout the genital tract and therefore constitutes a viable option for mutant complementation *in vivo*. Overall, the technological advances described here to generate conditional mutants in *Chlamydia* are simple to implement and will benefit the characterization of the role of *Chlamydia* effectors in cell culture and *in vivo* as the field moves forward.

One question that remains to be addressed is how to ascertain that IncS is essential, versus highly important. The *C*. *trachomatis ΔincS*_*Ct*_ mutant was severely attenuated in cell culture and *in vivo*; however, serial passages in the absence of aTc in cell culture did not result in sterilization, instead the *ΔincS*_*Ct*_ mutant produced consistent low level of infectious progeny at each passage (not shown). This result could be due to the fact that *incS* is highly important rather than essential; however, leakiness of the Tet inducible promoter could also explain this phenotype. We cannot at this point distinguish between these two possibilities. Essentiality is supported by the fact, that, after many failed attempts, allelic exchange and/or interruption of the *incS* ORF was only achieved in the presence of complementation, both in *C*. *trachomatis* and *C*. *muridarum*. Additionally, albeit non-saturating, chemical mutagenesis in *C*. *trachomatis* and transposition mutagenesis in *C*. *trachomatis* or *C*. *muridarum* did not result in premature stop codons in *incS*_*Ct*_ nor transposon insertion in *incS*_*Ct*_ or *incS*_*Cm*_ [26–28]. Moreover, although IncS-3xFLAG was not detected on the inclusion membrane in the absence of aTc, we cannot rule out that in some bacteria minute undetectable levels of IncS were sufficient to reach a certain threshold and rescued the developmental phenotype of the *incS* mutant. To settle *incS* essentiality, future studies are needed and will focus on improving the repression of the inducible promoter by combining aTc-dependent transcriptional regulation with a recently described riboswitch-dependent translational control [29]. A complementary approach will be to attempt to cure the complementation plasmid.

### IncS (CTL0402/CT147/TC0424)

IncS is an Inc protein conserved in all *Chlamydiae*, including *C*. *trachomatis* serovar L2 (CTL0402), serovar D (CT147), and *C*. *muridarum* (TC0424) [10, 13, 30, 31]. IncS is expressed 1h post-infection and throughout the developmental cycle [30].

A role for CT147 in inclusion avoidance of the endocytic pathway was proposed based on computational analysis and CT147 homology to the human Early Endosomal Antigen 1 (EEA1) protein [30]. The authors did not experimentally validate their hypothesis, and we did not observe any co-localization between *ΔincS*_*Ct*_ mutant inclusions and EEA1 or LAMP1. Additionally, using currently available online software, we were not able to identify any region of homology between EEA1 and CT147, or its homologues.

Recently, a role for CTL0402 and TC0424 in inclusion integrity and preventing host cell death was proposed based on *C*. *trachomatis/C*. *muridarum* chimeric strains, generated by lateral gene transfer, in which distinct regions of the *C*. *trachomatis* serovar L2 chromosome is replaced with region of the *C*. *muridarum* chromosome [31]. More specifically, replacement of *C*. *trachomatis ctl0402* with *C*. *muridarum tc0424* led to the loss of CTL0402 in the inclusion membrane, bacterial release into the host cell cytosol in mid-cycle, and an increase in the number of cells presenting hallmarks of apoptosis. Although strains with similar *C*. *trachomatis/C*. *muridarum* genetic make-up were compared and backcross experiments were performed, the phenotype was not complemented in trans, and it is unclear if the phenotype of the chimera is due to dysregulated TC0424 expression in a *C*. *trachomatis/C*. *muridarum* background.

Here using targeted mutagenesis and complemented strains, we were able to unequivocally link the genotype of *C*. *trachomatis* and *C*. *muridarum incS* mutants to a severe developmental defect in the early stages of the developmental cycle. Although *incS* mutant inclusions were negative for the endocytic pathway markers EEA1 and LAMP1, we cannot fully exclude mis-trafficking of the inclusions, as proposed by Belland et al. Similarly, although the early developmental defect of the *incS* mutants was not associated with mid-stage inclusion rupture or cell death, we cannot at this stage rule out that perturbation of inclusion membrane integrity early in the developmental cycle contributes to the phenotype of the *incS* mutant.

### Early *Chlamydia* development and EB to RB transition

The host and/or bacterial determinants that control each step of the *Chlamydia* developmental cycle, especially the EB to RB transition, are poorly characterized. The morphological difference between EB and RB is attributed to condensed and relaxed chromatin, respectively. The *Chlamydia* histone-like proteins Hc1 and Hc2 and a small metabolite of the non-mevalonate methylerythritol phosphate (MEP) pathway of isoprenoid biosynthesis are proposed to mediate DNA condensation [32, 33]. Proteomics profiling of EB and RB revealed significant differences, but none of these differences provided answers regarding the mechanism of EB to RB transition [34, 35]. Transcriptomic analysis of infected cells revealed that bacterial transcription is initiated as early as 1h pi and that bacterial genes are temporally expressed with transcription being initiated during the early (EB-RB transition), mid (RB replication) or late (RB-EB transition) stages of the developmental cycle [30, 36–41]. However, when investigating the genetic control of the initial step of the *Chlamydia* developmental cycle, there is very little, when any, overlap between the genes identified in the above referenced studies, most likely due to differences in approaches, timing of infection and normalization conditions. Additionally, except for the study by Rajeeve et al implicating glutamine uptake as a critical step for peptidoglycan synthesis and initiation of the EB conversion to RB [41], to date none of the candidates identified by transcriptomics have been validated to play a role in *Chlamydia* development in the context of infection, leaving the molecular mechanisms supporting EB to RB transition uncharacterized.

Our results indicate that IncS mutant bacteria are impaired in EB to RB transition during the early stages of *Chlamydia* development and that IncS function is conserved in *C*. *trachomatis* and *C*. *muridarum*. We do note that, under the conditions tested here, the *C*. *trachomatis incS*_*Ct*_ allele did not fully complement the phenotype of the *C*. *muridarum* mutant, perhaps because IncS_Ct_ and IncS_Cm_ only share 60% identity (76% similarity), which may result in suboptimal interaction with target protein(s). The partial complementation observed *in vivo* could also be due to the additive effect of a low final aTc concentration in the genital tract. Future studies will focus on elucidating how a single inclusion membrane protein contributes to successful progression through the early stages of the *Chlamydia* developmental cycle by potentially controlling the transcriptional reprogramming of infectious EB bacteria into replicative RB and/or remodeling the nascent inclusions into a replicative niche.

## Conclusion

In an attempt to overcome gene essentiality in obligate intracellular pathogens, we have adapted available genetic tools to generate conditional mutants in *C*. *trachomatis* and *C*. *muridarum*. The method for efficient *in vivo* complementation of a conditional mutant described here will be instrumental in applying the molecular Koch’s postulates when characterizing pathogenic properties of *Chlamydia* effector proteins [42]. Additionally, because *incS* is a *Chlamydia spp*.-specific gene expressed during human infections [43, 44] but absent from other bacterial species, including members of the microbiota, IncS is an ideal target for the development of antibiotics that would specifically inhibit *Chlamydia* development during infection.

## Material and methods

### Ethics statement

All genetic manipulations and containment work were approved by the University of Virginia (UVA) Biosafety Committee and are in compliance with the section III-D-1-a of the National Institute of Health guidelines for research involving recombinant DNA molecules. All animal experiments were approved by the UVA Institutional Animal Care and Use Committee.

### Cell lines and bacterial strains

HeLa cells (CCL-2) were obtained from ATCC and cultured at 37°C with 5% CO2 in high-glucose Dulbecco’s modified Eagle’s medium (DMEM; Invitrogen) supplemented with 10% heat-inactivated fetal bovine serum (Invitrogen). *C*. *trachomatis* lymphogranuloma venereum (LGV) type II was obtained from the ATCC (L2/434/Bu VR-902B). *C*. *muridarum* Nigg was obtained from Michael Starnbach (Harvard Medical School, Boston, MA). *C*. *trachomatis* and *C*. *muridarum* expressing mCherry under the control of the *groESL* promoter were described previously [21]. Chlamydia propagation, infection and transformation were performed as previously described [21, 45]. All *Chlamydia* strains were plaque purified.

### Plasmid construction

Restriction enzymes and T4 DNA ligase were obtained from New England BioLabs (Ipswich, MA). PCR was performed using Herculase DNA polymerase (Stratagene). PCR primers were obtained from Integrated DNA Technologies. Primers and cloning strategies are described in S1 Table and detailed below.

### Construction of pSU-ΔIncS

The plasmid was constructed via the Gibson assembly using HiFi DNA assembly master mix (New England Biolabs) following manufacturer instructions. 3-kb fragments downstream and upstream of *incS* (PCR A, right arm and PCR B, left arm, respectively) were amplified from *C*. *trachomatis* L2 genomic DNA via PCR using primers pSUmC3Dwn0402 5 2.1 and 3Dwn0402pSumC 3 2.1 and pSUmC3Up0402 5 and 3Up0402pSUmC 3, respectively. PCR A and PCR B were sequentially cloned into the SbfI and SalI sites of pSUmC-4.0, respectively, so that each arm immediately flanked the *aadA-gfp* cassette [46].

### Construction of pSW2-TetIncS_Ct_

DNA fragments corresponding to the *tet* repressor (*tetR*), *tetA* promoter (*tetA*^*P*^) (PCR A) and to the 3xFLAG and the *incDEFG* operon terminator (PCR C) were amplified by PCR from p2TK2-SW2 mCh(Gro) Tet-IncV-3F plasmid [47] using primers TetRSTOP5Kpn and TetAP-IncS Rv, and 0402FLAG Fw and IncDTerm3Not, respectively. A DNA fragment corresponding to the *ctl0402* ORF (PCR B) was amplified from *C*. *trachomatis* L2 genomic DNA by PCR using primers TetAP-IncS Fw and 0402 FLAG Rv. A DNA fragment corresponding to TetR-tetAP CTL0402 3xFLAG *incDEFG* terminator (PCR D) was amplified by overlapping PCR using PCR A, B and C as template and primers TetRSTOP5Kpn and IncDTerm3Not. A DNA fragment (PCR E) corresponding to *Neisseria meningitidis* promoter-GFP (nmP-GFP) was amplified by PCR from pGFP::SW2 [48] with primers AgeI nmP Prom Fw and RSGFP TAA AgeI Rv. Fragment D and E were sequentially cloned into the KpnI/NotI sites and AgeI site, respectively, of p2TK2_Amp_-SW2 [49].

### Construction of pNigg-TetIncS_Ct_

The DNA fragment corresponding to TetR-tetA^P^ CTL0402 3xFLAG *incDEFG* terminator was obtained by KpnI/NotI digest of pSW2-TetIncS_Ct_ and cloned into p2TK2_Spec_-Nigg mCh(Gro_L2_) [21].

### Construction of pDFTT3-IncS_Cm_

The GrpII intron was retargeted for *tc0424* (IncS_Cm_) using primers TC0424-412 IBS1/2, TC0424-412 EBS1/delta, and TC0424-412 EBS2 designed by the TargeTron computer algorithm (TargeTronics). The resulting PCR product was digested with BsrGI and HindIII and cloned into the BsrGI/HindIII site of the pDFTT3-*bla-*IncA suicide vector [19].

### Validation of *Ct* ΔincS_Ct_ pTet-IncS_Ct_

**Allelic exchange** of the *incS* ORF with the *aadA-gfp* cassette was confirmed by PCR (S1 Fig and S1 Table). WT and *ΔincS*_*Ct*_ pTet-IncS_Ct_ genomic DNA, prepared using illustra bacteria genomicPrep Mini Spin Kit (GE Healthcare), according the manufacturer recommendations, were used as template in PCR reactions using two sets of primers surrounding the 3kb homology recombination site (incS 3kb Up Fw & incS 3kb Dwn Rv) or surrounding the *incS* ORF (incS Up Fw & incS Dwn Rv). The PCR products were run into 1% agarose gel, stained with ethidium bromide, and viewed using UV transillumination. The PCR products were extracted from the agarose gel using QIAquick Gel Extraction Kit (Qiagen) and sequenced via Sanger performed by Genscript.

**The conditional expression and inclusion localization of IncS**_**Ct**_ was validated by immunofluorescence. HeLa cells seeded on glass coverslips were infected with *Ct* ΔincS_Ct_ pTet-IncS_Ct_ in the presence of 0.5ng/ml aTc (+aTc) or not (-aTc) for 24 h, fixed stained with mouse monoclonal anti-FLAG (1:1,000; Sigma) and secondary antibodies (Alexa Fluor 594-conjugated goat anti-mouse antibody (1:500; Molecular Probes) and processed for confocal microscopy.

### Validation of *Cm incS*_*Cm*_::*bla* pTet-IncS_Ct_

**Intron integration** and interruption of the *tc0424* (*incSC*_*m*_) ORF was confirmed by PCR using genomic DNA and primers TC0424 Ups Fw and TC0424 (691–719) Rv, which flank the insertion site of the intron (S4 Fig and S1 Table). The PCR products amplified from mutant genomic DNA showed the expected 2 kb increase in band size compare to wild-type.

**The conditional expression and inclusion localization of IncS**_**Ct**_ was validated by immunofluorescence as described for *Ct* ΔincS_Ct_ pTet-IncS_Ct_, except that infection was performed in the presence of 1ng/ml aTc.

### Immunofluorescence and confocal microscopy

All steps were performed at room temperature. At the indicated times, cells were fixed with 4% paraformaldehyde in 1× PBS for 30 min and sequentially incubated with primary and secondary antibodies diluted in 0.1% Triton X-100 in 1× PBS for 1h or with the DNA dye 7AAD-red. Coverslips were washed with 1× PBS and mounted with DABCO antifade-containing mounting medium. Confocal microscopy was performed using the Leica DMi8 microscope equipped with the Andor iXon ULTRA 888BV EMCCD camera and the confocal scanner unit CSU-W1, and driven by the IQ software. Images were processed using the Imaris software (Bitplane, Belfast, United Kingdom).

### Infectious progeny production

HeLa cells seeded in 96 well plates were infected with the *C*. *trachomatis* or *C*. *muridarum incS* conditional mutant at an MOI of 0.5 in medium containing 0.5ng/ml of aTc or at an MOI of 10 without aTc. Infection was synchronized by centrifugation for 10 min at 600g. Cells were lysed at 0h and 48h (*C*. *trachomatis*) or 39h (*C*. *muridarum*) post infection in 100μl sterile water, and 5-fold dilutions of the lysates were used to infect fresh HeLa cell monolayers seeded in 384 well plates in the presence of aTc. At 24h pi cells were fixed with 4% paraformaldehyde in 1x PBS. Infected cells were stained with Hoechst. Quantification of inclusion forming units (IFUs) per ml using the MetaXpress software. Fold growth was determined as the ratio between IFUs/ml at 48h (*C*. *trachomatis*) or 39h (*C*. *muridarum*) pi/0h pi.

### Percentage of cells with an inclusion upon addition or removal of aTc at different time post infection

HeLa cells seeded in 96 well black plates were infected with the *C*. *trachomatis ΔincS*_*Ct*_ conditional mutant at an MOI of 1 in the absence (Fig 2A) or the presence of 0.5ng/ml of aTc (Fig 2B). Infection was synchronized by centrifugation for 10 min at 600g. Cells were washed 5 times with 200 μl of DMEM at 2, 4, 6 or 8h pi and the medium was replaced with 0.5ng/ml aTc (Fig 2A) or without aTc (Fig 2B). The cells were fixed with 4% paraformaldehyde in 1x PBS at 28h pi. Infected cells were stained with Hoechst. The percentage of infection was determined using the MetaXpress software.

### Intracellular localization and morphology of the *C*. *trachomatis incS* conditional mutant

HeLa cells seeded on glass coverslips were infected with the *C*. *trachomatis ΔincS*_*Ct*_ conditional mutant at an MOI of 10 in the presence of 0.5ng/ml aTc, absence aTc, or with aTc added at 4h pi. Infection was synchronized by centrifugation for 10 min at 600g. At 2h pi cells were washed 5 times with 500 μl of DMEM to remove the non-infectious bacteria and the medium was replaced. At 12h pi samples were fixed with 4% paraformaldehyde stained with the DNA dye 7AAD-red and processed and imaged by confocal microscopy. The number of total intracellular bacteria (as determined by bacteria that were in the same focal plane as the nuclei) and RB (as determine by bacteria of about 1 μm in diameter with the Imaris software) per cell were quantified manually with the Imaris Imaging software. 150 infected cells were analyzed per sample.

### Electron microscopy

HeLa cells were infected with the *C*. *trachomatis ΔincS*_*Ct*_ conditional mutant in the presence of 0.5ng/ml aTc (+aTc) or not (-aTc) at an MOI of 15. Infection was synchronized by centrifugation for 10 min at 600g. Twelve hours post-infection, the samples were fixed in 2.5% glutaraldehyde in 0.1 M cacodylate buffer (pH 7.4) for 60 min at RT. After fixation, cells were scraped off and pelleted, as described previously [50]. Ultrathin sections of infected cells were stained with osmium tetraoxide before examination with Hitachi 7600 EM under 80 kV equipped with a dual AMT CCD camera system.

### Quantification of OmcA levels

HeLa cells seeded on coverslips were infected with the *C*. *trachomatis ΔincS*_*Ct*_ conditional mutant at an MOI of 15 in the presence of 0.5ng/ml aTc or absence of aTc. At 6h and 12 hpi, the cells were processed for immunofluorescence and confocal microscopy as described above, except that 0.2% Triton X-100 was used for permeabilization and immunostaining. OmcA was detected using rabbit polyclonal anti-OmcA antibodies [21] (1 in 200 in 0.2% Triton X-100 in 1× PBS over-night at 4°C), followed by Alexa Fluor 594-conjugated goat anti-rabbit antibody (1:500; Molecular Probes) and the DNA dye SytoxGreen (1:50,000; Invitrogen), both diluted in 0.1% Triton X-100 in 1× PBS and incubated at room temperature for 1h. After confocal imaging, the levels of OmcA associated with perinuclear bacteria were quantified as described below and in S3 Fig. The baseline background was subtracted for each channel (green for DNA, red for OmcA) (Step 1). For each infected cell, a surface area containing the bacteria clustered at the perinuclear region of the cell was manually created in every other XY plane of merge images (Step 2). A 3D object combining the surface areas from step 2, and the intercalating XY planes, was generated (Step 3). The sum intensity of the OmcA and DNA signal within the 3D object from step 3 was recorded (Step 4). The OmcA levels, in arbitrary units, were determined by normalizing the sum intensity of the OmcA signal with the sum intensity of DNA signal (Step 5). For each condition, a total of 30 infected cells were quantified. Infected cells where the bacteria were overlapping with the host nucleus or with another infected cell were excluded from the analysis.

### Intravaginal infections and vaginal shedding

Six-to-eight-week-old female C3H/HeJ mice (The Jackson Laboratory) were treated subcutaneously with 2.5 mg medroxyprogesterone acetate (Depo-Provera, Pfizer) 7 days prior to infection. When indicated, aTc (10μg/ml final) was added to the drinking water 4 days prior to infection and for the duration of the experiment. aTc treated water was kept in amber bottles and replaced every other day. In a pilot experiment, final concentration of aTc in the drinking water of 100μg/ml or 10μg/ml resulted in similar complementation. Testing higher concentrations were cost-prohibitive (at least when providing fresh water every other day). We did not test lower concentrations or try replacing the water less frequently. To monitor vaginal shedding, 5x10^5^ IFUs of the indicated *C*. *muridarum* strain were introduced in the vaginal vault (5 mice per group). Shedding was monitored every other day by swabbing the vaginal vault. Each swab was collected in 500 μl of cold SPG with glass beads and vortexed vigorously. The released bacteria were serially diluted in SPG and titrated on HeLa cell monolayers in medium containing 2.5 μg/ml Amphotericin B (Gibco), 50 μg/ml gentamycin (Gibco), and 1 ng/ml aTc. At 18h pi, the number of inclusions for each condition was enumerated based on mCherry expression and used to calculate log IFUs/ml. Mice were swabbed until the infection was cleared, as determined by 2 consecutive negative swabs.

### Transcervical infections and tissue preparation for microscopy

To visualize *C*. *muridarum* infected cells *in vivo*, 5x10^5^ IFUs were delivered directly into the uterine horn of six-to-eight-week-old female C3H/HeJ mice (The Jackson Laboratory), using a non-surgical embryo transfer device (ParaTechs Corp) (3 mice per group). Mice were pre-treated with 2.5 mg medroxyprogesterone acetate (Depo-Provera, Pfizer) 7 days prior to infection. When indicated, aTc was added to the drinking water as described for intravaginal infections. The mice were sacrificed at 8h pi. The genital tracts were excised and placed into histology cassettes. The cassettes were immersed in 10% formalin for 48h. The tissues were preserved in 70% ethanol before loading onto a tissue processor for dehydration and paraffin infiltration. After manual embedding into a paraffin block, paraffin sections were cut at 5 μm on a Leica microtome.

### Microscopy of infected tissue

Paraffin sections were deparaffinized and dehydrated in the following sequence: xylene, 100% ethanol, and hydrogen peroxide. Antigen retrieval was performed at 95–100°C for 15 min using boiling 1x RNAscope Target Retrieval Reagent (Advanced Cell Diagnostics, 322000). Slides were rinsed in deionized water for 15 seconds and washed in 100% ethanol for 3 min.

Slides were permeabilized with RNAscope Protease Plus for 30 min at 40°C within the RNAscope HybEZ II Hybridization System. Slides were washed in RNAscope Wash Buffer (Advanced Cell Diagnostics, 310091). The *C*. *muridarum*-23srRNA-C1 probe (Advanced Cell Diagnostics, 1039531-C1) was hybridized on the slides for 2 h at 40°C within the RNAscope HybEZ II System. Slides were washed in RNAscope Wash Buffer. Slides were incubated within the RNAscope HybEZ II System using the RNAscope Multiplex Fluorescent Detection Reagents v2 kit (Advanced Cell Diagnostics, 323110) in order at 40°C: AMP 1 (Advanced Cell Diagnostics, 323101) for 30 min, AMP 2 (Advanced Cell Diagnostics, 323102) for 30 min, AMP 3 (Advanced Cell Diagnostics, 323103) for 15 min, HRP-C1 (Advanced Cell Diagnostics, 323104) for 15 min, Opal 690 Reagent (Akoya Biosciences, OP-001006) at 1:1000 within RNAscope Multiplex TSA Buffer (Advanced Cell Diagnostics, 322809) for 30 min, and HRP blocker (Advanced Cell Diagnostics, 323107) for 15 min. After each incubation step, slides were washed in RNAscope Wash Buffer for 5 min with gentle agitation.

Immediately following RNAscope procedure, slides were blocked with 2% normal goat serum in 5% Bovine Serum Albumin (AmericanBio, AB01088-00100) PBS for 1 h at room temperature. Primary antibodies were diluted 1:100 in blocking buffer (E-cadherin, BD Biosciences 610181) and incubated overnight at RT. Secondary antibodies (goat anti-mouse Alexa Fluor 488,) were diluted 1:500 in blocking buffer and incubated for 2 h at room temperature. Slides were counterstained using RNAscope Multiplex FL v2 DAPI (Advanced Cell Diagnostics, 323108) for 1 min. Coverslips were mounted using ProLong Gold Antifade Mountant (Thermo Fisher Scientific, P36930). Slides were imaged using a Nikon TE2000 microscope equipped for automated multi-color imaging including motorized stage and filter wheels, a Hamamatsu Orca ER Digital CCD Camera and piezo-driven ×10 objective. The corresponding images were processed with the MetaMorph software (Molecular Devices, Inc.).

### Statistics

Except when noted, each experiment was performed in triplicate, and the average and SEM from one representative experiment are shown. The graphs were generated using GraphPad Prism. The appropriate statistical tests were used and are indicated in the Figure legends.

## Supporting information

S1 Fig Validation of the *Chlamydia trachomatis incS* conditional mutant. (A) Schematic representation of the *incS*_*Ct*_ locus of wild-type (WT) and Δ*incS*_*Ct*_ mutant *C*. *trachomatis* strains and of the primers used for mutant validation by PCR. P1 (incS 3kb Up Fw), P2 (incS 3kb Dwn Rv), P3 (incS Up Fw), P4 (incS Dwn Rv). (B) DNA gels of PCR products generated using the following combination of genomic DNA template/primer, as described in (A). Left panel: WT/P1P2 (lane 2) and Δ*incS*_*Ct*_/P1P2 (lane 3). Right panel: WT/P3P4 (lane 2) and Δ*incS*_*Ct*_/P3P4 (lane 3). The ladder is shown in lane 1.
(TIF)

S2 Fig Ultrastructural analysis of *Chlamydia trachomatis incS* conditional mutant bacteria. Transmission electron micrographs of sections of HeLa cells infected with a *C*. *trachomatis* Δ*incS*_*Ct*_ *conditional* mutant at an MOI of 15 for 12h in the presence (+aTc) or absence (-aTc) of aTc. RB: Reticulate Body; IB: Intermediate Body. Scale bar: 1 μm.
(TIF)

S3 Fig Method to quantify the levels of OmcA associated with perinuclear bacteria. Step 1: The baseline background was subtracted for each channel (green for DNA, red for OmcA) Step 2: For each infected cell, a surface area containing the bacteria clustered at the perinuclear region of the cell was manually created in every other XY plane. Step 3: A 3D object was generated combining the surface areas from step 2. Step 4: The sum intensity of the OmcA and DNA signal within the 3D object from step 3 was recorded: Step 5: The OmcA levels, in arbitrary units, was determined by normalizing the Sum intensity of the OmcA signal with the Sum intensity of DNA signal.
(TIF)

S4 Fig Validation of the *Chlamydia muridarum incS* conditional mutant. (A) Schematic representation of the *incS*_*Cm*_ locus of wild-type (WT) and *incS*_*Cm*_::*bla* mutant *C*. *muridarum* strains, and of the primers used for mutant validation by PCR. P1 (TC0424 Up Fw), P2 (TC0424 (691–719)). (B) DNA gels of PCR products generated using the following combination of genomic DNA template/primer, as described in (A). WT/P1P2 (lane 2) and *incS*_*Cm*_::*bla*/P1P2 (lane 3). The ladder is shown in lane 1. (C) The site of insertion of the group II intron was confirmed by Sanger sequencing. A translation of the resulting IncSCm truncated peptide is presented. The asterisk denotes the early stop codon introduced by the insertion of the group II intron (brown: IncS_Cm_, blue: group II intron).
(TIF)

S1 Table Primers pairs (name and sequence) and corresponding templates used in this study. (XLSX)

S1 Data set Raw data from Fig 1. (XLSX)

S2 Data set Raw data from Fig 2. (XLSX)

S3 Data set Raw data from Fig 3. (XLSX)

S4 Data set Raw data from Fig 4. (XLSX)
