# Supplementary figures and images for: The inclusion membrane protein IncS is critical for initiation of the Chlamydia intracellular developmental cycle
Source: PLoS Pathog. 2022 Sep 9;18(9):e1010818. doi: 10.1371/journal.ppat.1010818 (PMC9491573; doi:10.1371/journal.ppat.1010818)

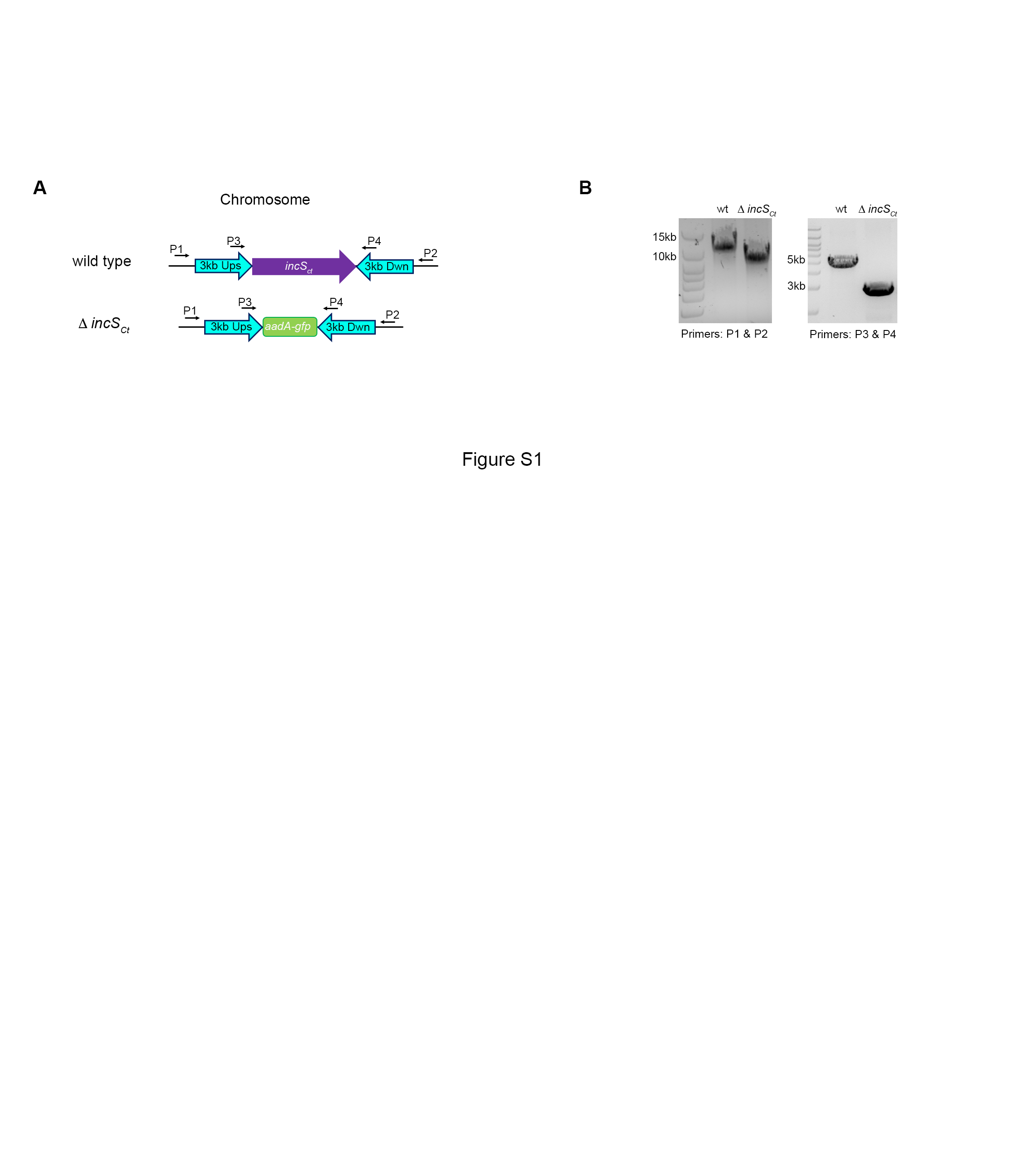

Supplement: S1 Fig — (A) Schematic representation of the incSCt locus of wild-type (WT) and ΔincSCt mutant C. trachomatis strains and of the primers used for mutant validation by PCR. P1 (incS 3kb Up Fw), P2 (incS 3kb Dwn Rv), P3 (incS Up Fw), P4 (incS Dwn Rv). (B) DNA gels of PCR products generated using the following combination of genomic DNA template/primer, as described in (A). Left panel: WT/P1P2 (lane 2) and ΔincSCt/P1P2 (lane 3). Right panel: WT/P3P4 (lane 2) and ΔincSCt/P3P4 (lane 3). The ladder is shown in lane 1. (TIF) [file ppat.1010818.s001.tif]

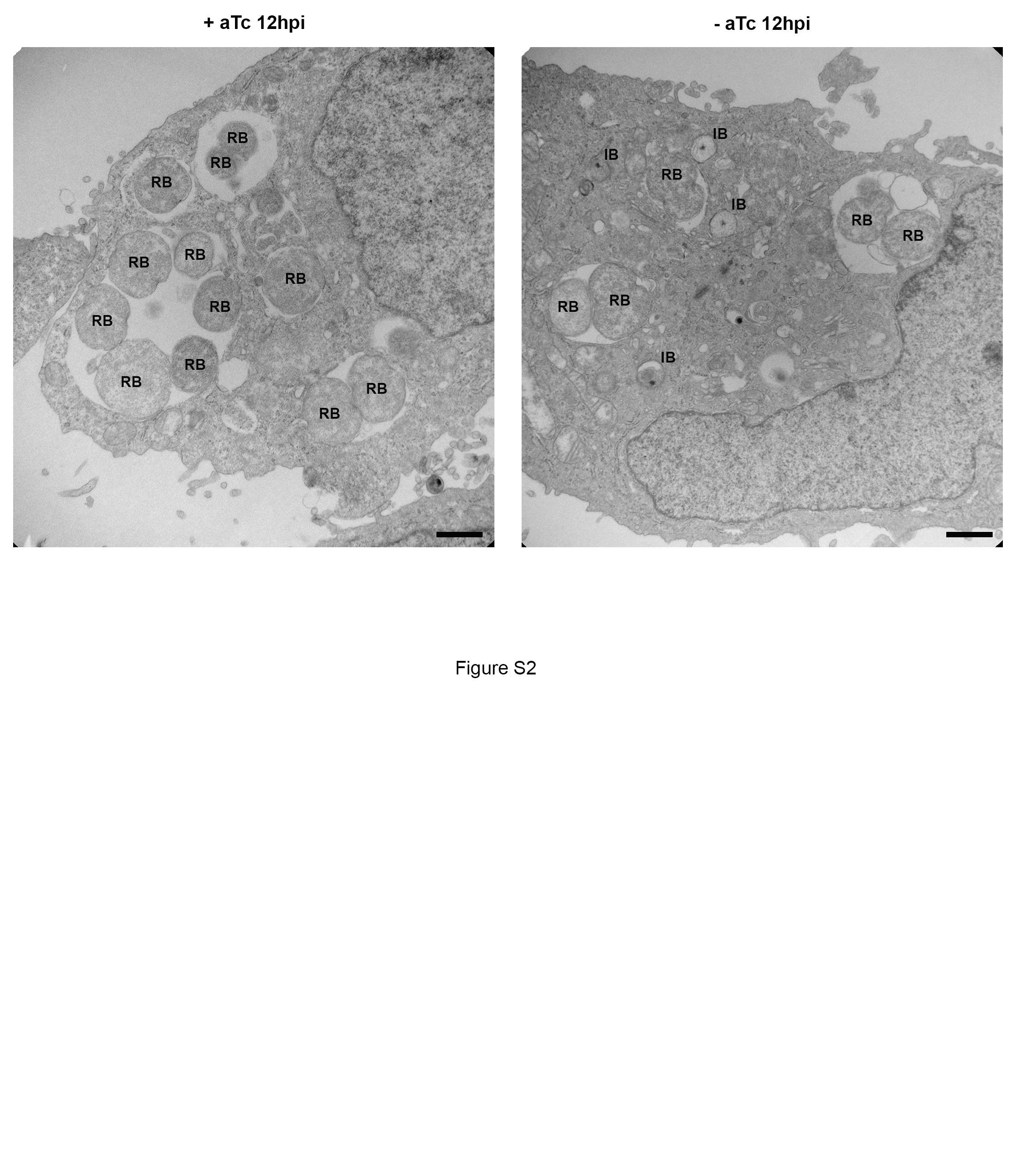

Supplement: S2 Fig — Transmission electron micrographs of sections of HeLa cells infected with a C. trachomatis ΔincSCt conditional mutant at an MOI of 15 for 12h in the presence (+aTc) or absence (-aTc) of aTc. RB: Reticulate Body; IB: Intermediate Body. Scale bar: 1 μm. (TIF) [file ppat.1010818.s002.tif]

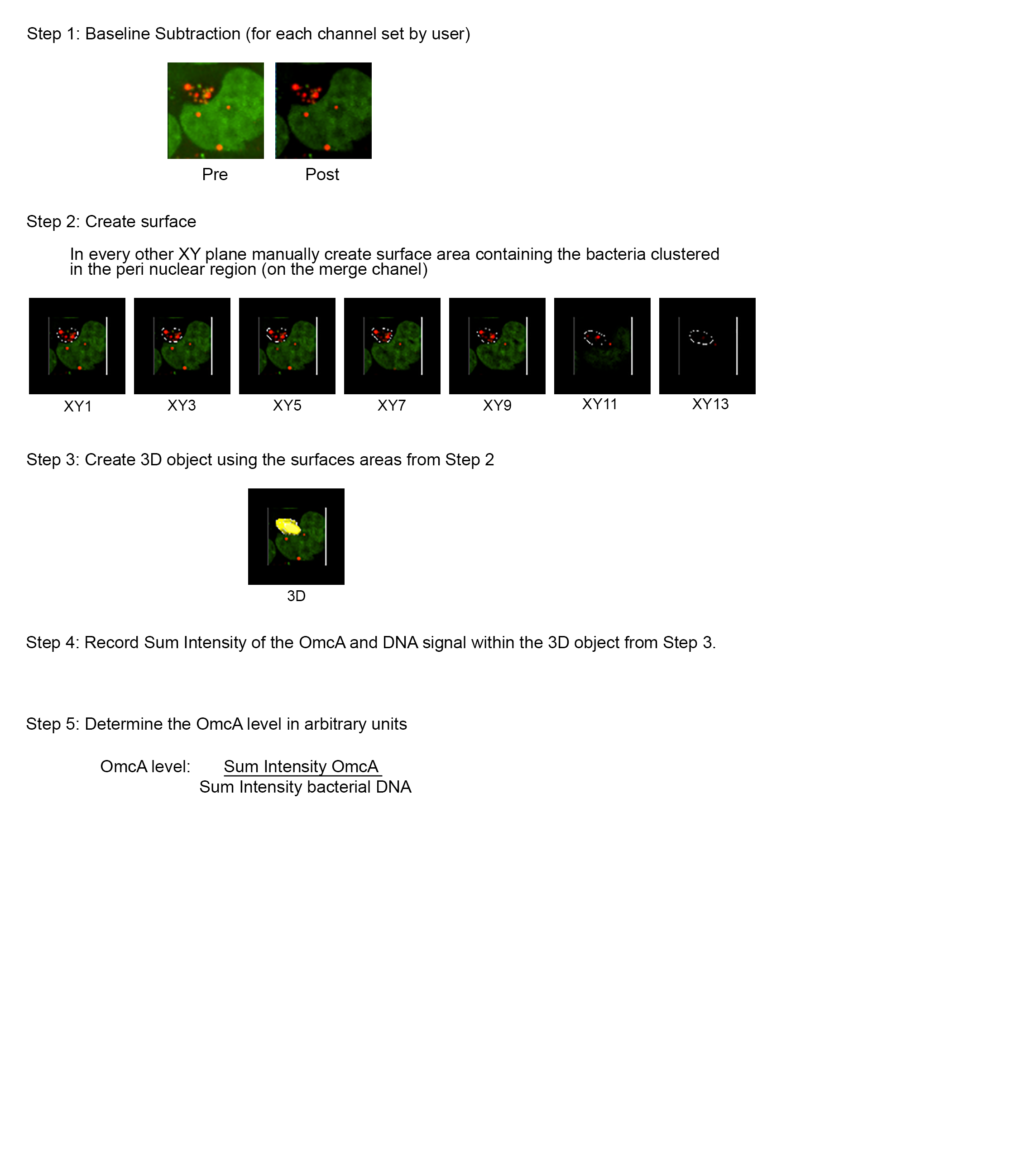

Supplement: S3 Fig — Step 1: The baseline background was subtracted for each channel (green for DNA, red for OmcA) Step 2: For each infected cell, a surface area containing the bacteria clustered at the perinuclear region of the cell was manually created in every other XY plane. Step 3: A 3D object was generated combining the surface areas from step 2. Step 4: The sum intensity of the OmcA and DNA signal within the 3D object from step 3 was recorded: Step 5: The OmcA levels, in arbitrary units, was determined by normalizing the Sum intensity of the OmcA signal with the Sum intensity of DNA signal. (TIF) [file ppat.1010818.s003.tif]

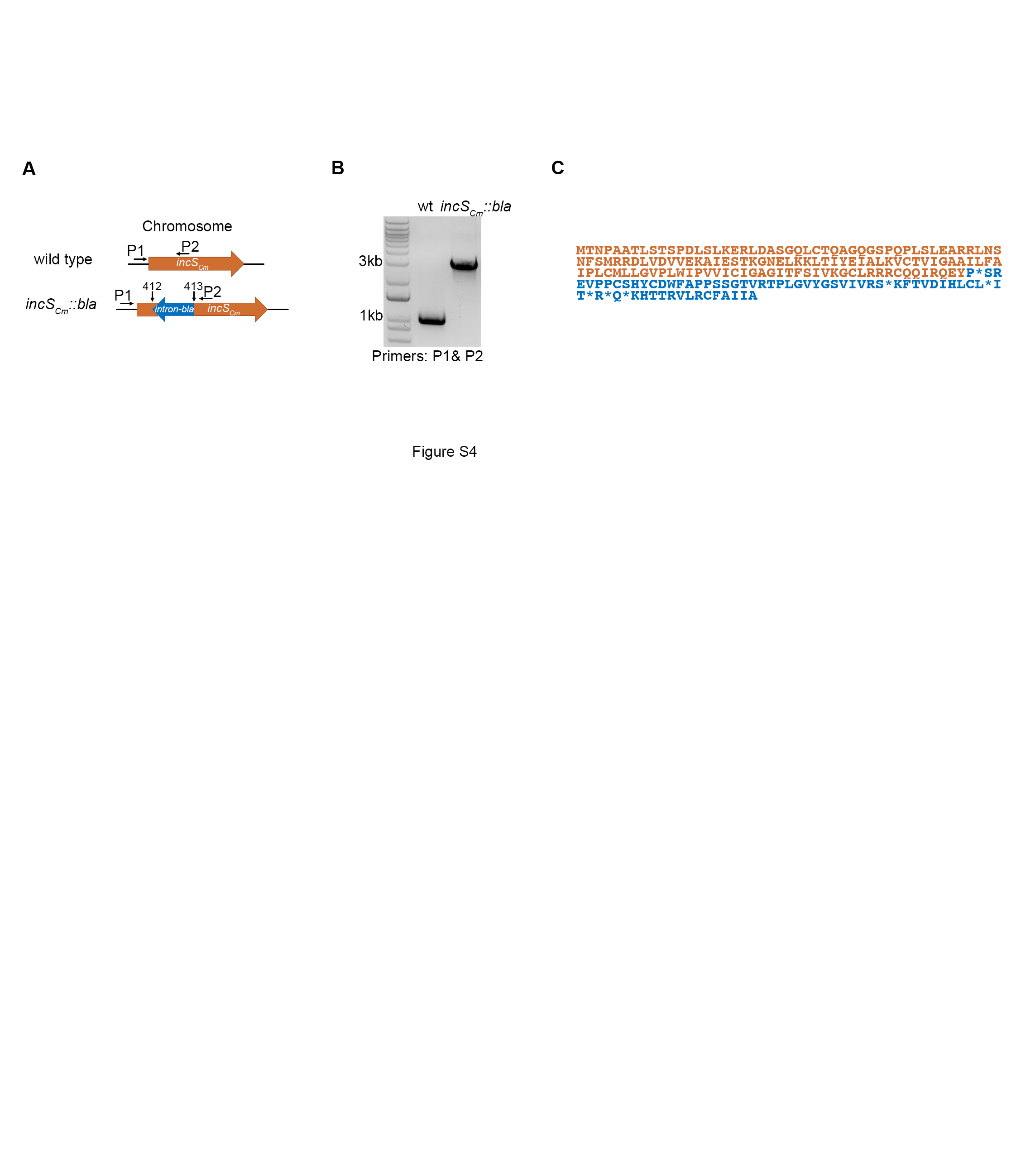

Supplement: S4 Fig — (A) Schematic representation of the incSCm locus of wild-type (WT) and incSCm::bla mutant C. muridarum strains, and of the primers used for mutant validation by PCR. P1 (TC0424 Up Fw), P2 (TC0424 (691–719)). (B) DNA gels of PCR products generated using the following combination of genomic DNA template/primer, as described in (A). WT/P1P2 (lane 2) and incSCm::bla/P1P2 (lane 3). The ladder is shown in lane 1. (C) The site of insertion of the group II intron was confirmed by Sanger sequencing. A translation of the resulting IncSCm truncated peptide is presented. The asterisk denotes the early stop codon introduced by the insertion of the group II intron (brown: IncSCm, blue: group II intron). (TIF) [file ppat.1010818.s004.tif]
